# Biomolecular Interaction Analysis Using an Optical Surface Plasmon Resonance Biosensor: The Marquardt Algorithm vs Newton Iteration Algorithm

**DOI:** 10.1371/journal.pone.0132098

**Published:** 2015-07-06

**Authors:** Jiandong Hu, Liuzheng Ma, Shun Wang, Jianming Yang, Keke Chang, Xinran Hu, Xiaohui Sun, Ruipeng Chen, Min Jiang, Juanhua Zhu, Yuanyuan Zhao

**Affiliations:** 1 Department of Electrical Engineering, Henan Agricultural University, Zhengzhou, China; 2 State Key Laboratory of Wheat and Maize Crop Science, Zhengzhou, China; 3 School of Materials Science and Engineering, Shanghai University, Shanghai, China; 4 School of Human Nutrition and Dietetics, McGill University, Ste Anne de Bellevue, Quebec, Canada; 5 College of Life Sciences, Henan Agricultural University, Zhengzhou, China; 6 Hanan Mechancial and Electrical Vocational College, Zhengzhou, China; CNR, ITALY

## Abstract

Kinetic analysis of biomolecular interactions are powerfully used to quantify the binding kinetic constants for the determination of a complex formed or dissociated within a given time span. Surface plasmon resonance biosensors provide an essential approach in the analysis of the biomolecular interactions including the interaction process of antigen-antibody and receptors-ligand. The binding affinity of the antibody to the antigen (or the receptor to the ligand) reflects the biological activities of the control antibodies (or receptors) and the corresponding immune signal responses in the pathologic process. Moreover, both the association rate and dissociation rate of the receptor to ligand are the substantial parameters for the study of signal transmission between cells. A number of experimental data may lead to complicated real-time curves that do not fit well to the kinetic model. This paper presented an analysis approach of biomolecular interactions established by utilizing the Marquardt algorithm. This algorithm was intensively considered to implement in the homemade bioanalyzer to perform the nonlinear curve-fitting of the association and disassociation process of the receptor to ligand. Compared with the results from the Newton iteration algorithm, it shows that the Marquardt algorithm does not only reduce the dependence of the initial value to avoid the divergence but also can greatly reduce the iterative regression times. The association and dissociation rate constants, *k_a_*, *k_d_* and the affinity parameters for the biomolecular interaction, *K_A_*, *K_D_*, were experimentally obtained 6.969×10^5^ mL·g^-1^·s^-1^, 0.00073 s^-1^, 9.5466×10^8^ mL·g^-1^ and 1.0475×10^-9^ g·mL^-1^, respectively from the injection of the HBsAg solution with the concentration of 16ng·mL^-1^. The kinetic constants were evaluated distinctly by using the obtained data from the curve-fitting results.

## Introduction

Kinetic analysis of biomolecular interactions that are affected by partial mass transfer is the most difficult task in the quantity of binding kinetic constants [1–2]. There are several approaches have been suggested to perform the analysis of biomolecular interactions based on the use of the kinetic model, differing in how data are selected [3]. The high quality kinetic data can be extracted from the curve where binding is closer to equilibrium. The kinetic analysis of biomolecular interaction can also be applied to detect the toxic molecules in food safety, environmental pollutants and life science [4–5]. In immunology, the binding strength between antibody and antigen reflects the biological activities of the control antibody and its immune response significance in the pathologic process [6–7]. Moreover, both the association rate and dissociation rate of antigen to antibody (receptor to ligand) are the important parameters for the study of signal transfer between cells [8–9]. The traditional methods involving the kinetic analysis of biomolecular interactions are mainly included ELISA (Enzyme-Linked Immunosorbent Assay), Equilibrium Dialysis, and Affinity Chromatography [10–12]. By comparison with these technologies, the most prominent characteristics of the surface plasmon resonance biosensor are the real-time monitor of the kinetic process without marking the biological molecules. In these experiments of biomolecular interactions using the traditional method, a limited binding partner with suitable spectroscopic properties such as fluorescent tags or other labels should be screened to give a useful fluorescence spectrum [13]. However, in recent advances, the development of optical surface plasmon resonance (SPR) biosensors for accurate kinetic and affinity analysis makes the biomolecular monitor powerful [14–16]. The ability of SPR biosensors to analyze biomolecular interactions in real time features the quantity of the affinity of ligand for its receptor and the kinetics parameters of the interaction [17]. Furthermore, SPR technology makes possible a detailed analysis of biomolecular interactions at the molecular level, as well as enabling the analysis of multimolecular complex assembly and function [18–19]. From the sensorgram obtained from the SPR biosensor, the relationship between RUs (response unit) and time(s) established in the process of association and dissociation of biomolecular interactions is a complicated nonlinear function. There is a number of instrumentation-based analysis software for use in obtaining kinetic constants. For example, sensorgrams may be figured out using one of several binding models provided with evaluation software from SensiQ and Autolab [20]. Another analysis approach is performed by using the powerful software OriginPro which can be applied to process the known data using the nonlinear curve fitting. Although the kinetic constants can be obtained easily from the known evaluation software, however, it can’t be embedded into the microcontroller in the design of the homemade bioanalyzer using the SPR biosensor due to intellectual property. The Newton iteration algorithm for the calculation of kinetic constants of biomolecular interactions has been described [21]. We have found that this method had a great dependence of the initial value. Moreover, the fitting results are likely to be divergent with different initial conditions. This paper proposes a powerful method to implement the kinetic data analysis for biomolecular interactions using the Marquardt algorithm based on Gauss-Newton algorithm. The pseudo first order kinetic model of biomolecular interaction was established firstly. Then, the data collected from the biomolecular interaction between the hepatitis B surface antigen (HBsAg) and the hepatitis B surface antibody (HBsAb) was obtained by the homemade SPR bioanalyzer [22]. Finally, we used this approach established by the Marquardt algorithm to perform the nonlinear curve-fitting for the calculation of the association and dissociation rate constants and the affinity constants. The results show that Marquardt algorithm does not only reduce the dependence of initial value to avoid the problem of data divergence but also greatly reduce the iterative regression times.

## Materials and Methods

### Materials

The three-channel Spreeta modules (TSPR1K23) manufactured with the gold slide bonded to the sensor modules were from Nomadics, Inc. (Stillwater, USA). Hepatitis B surface antibody (HBsAb) was purchased from Zhengzhou Biocell Antibody Centre (Henan, China). The HBsAb was stored frozen, and its standard solutions were prepared daily with phosphate buffer solution (PBS). An ELISA diagnostic kit for Hepatitis B surface antigen (HBsAg) was purchased from Shanghai Rongsheng Biotech Co., Ltd. (Shanghai, China). The standard HBsAg solutions were diluted with PBS (pH 7.4) and stored at 4°C. Double distilled water was used throughout the whole experiment. A 0.01M PBS (pH 7.4) was prepared by dissolving 0.24 g KH_2_PO_4_, 8.0 g NaCl, 1.44 g K_2_HPO_4_ and 0.2 g KCl in 1000 mL double distilled water.

### Kinetic model

If a single ligand binds to the receptor in a 1:1 stoichiometric ratio to form the receptor-ligand complexes, the association process is described by considering two substances ligand *L* and receptor *R*, which were combined to emerge a complex *LR* [23–25]. In a practical reaction, both the association and dissociation processes occur simultaneously. For reversible associations and dissociations in a chemical equilibrium, it can be described by the following expression:

$$L+R\overset{k_{a}}{\underset{k_{d}}{⇌}}LR$$ (1)

where, *k*_*a*_ (mol•L^-1^•s^-1^) is the association rate constant used to describe the binding kinetic constant between ligand *L* and receptor *R*. The dissociation rate constant *k*_*d*_ (s^-1^) is the ratio of the concentration of the dissociated complex to the undissociated complex.

It is equally valid to write the rate equations as follows:

$$\text{Association rate:}\frac{d[LR]}{dt}=k_{a}C_{L}C_{R}$$ (2)

$$\text{Dissociation rate:}-\frac{d[LR]}{dt}=k_{d}C_{LR}$$ (3)

$$\text{Net rate equation:}\frac{d[LR]}{dt}=k_{a}C_{L}C_{R}-k_{d}C_{LR}$$ (4)

where the brackets denote concentrations of the free *R*, free *L* and the concentrations of the complex *[RL]* at equilibrium. From this equation, it can be seen that dissociation rate *k*_*d*_ and association rate *k*_*a*_ for a given system can be determined any time. The concentrations of *[R]*, *[L]*, and *[RL]* are measured under equilibrium conditions.

The net rate reached approximately to zero when the equilibrium condition was formed. That is $\frac{d[LR]}{dt}=0$ and *k*_*a*_ *C*_*L*_ *C*_*R*_ = *k*_*d*_ *C*_*LR*_, therefore, it can be already expressed as

$$\frac{k_{a}}{k_{d}}=\frac{C_{LR}}{C_{L}C_{R}}=K_{A}=\frac{1}{K_{D}}$$ (5)

where, *K*_*A*_ and *K*_*D*_ are the equilibrium association and dissociation constants.

In the ligand binding process, two reactions take place as follows: (a) the total number of associations per unit time interval in a particular region is proportional to the total number of receptors involved, because they all can create a complex with the same probability [26–27]. The relationship among the amount of the complexes formed per unit time *Ca*, the instantaneous concentration of the free analyte *C*_*L*_, and the concentration of free receptors *C*_*R*_ *-C*_*LR*_ is expressed as

$$\frac{dC_{a}}{dt}=k_{a}C_{L}(C_{\text{R}}-C_{\text{LR}})$$ (6)

(b) on the other hand, for each compound, there is certain probability that it will be dissociated into ligand *L* and receptor *R* within a unit time interval. This probability is the same for all compounds at the given conditions. The dissociation leads to a decrease of the compound concentrations proportional to its instantaneous value described as:

$$\frac{dC_{\text{d}}}{dt}=-k_{d}C_{\text{LR}}$$ (7)

where, *C*_*d*_ is the amount of the complex *LR* associated per unit time.

The rate of consumption of ligand *L* depends on both the concentration of ligand *L* and the concentration of receptor *R*. The chemical equilibrium Eq (1) can be expressed by the pseudo first order reaction rate equation (Kinetic equation) [28]. The corresponding differential equation is derived as follows:

$$\frac{dC_{LR}}{dt}=\frac{dC_{a}}{dt}+\frac{dC_{\text{d}}}{dt}=k_{a}C_{L}(C_{R}-C_{LR})-k_{d}C_{LR}$$ (8)

The instantaneous concentrations of complex *LR* can be indicated by the response values (*R*) of the SPR biosensor. Furthermore, the concentrations of unfree receptor *R* obtained at equilibrium are represented by *R*_*max*_, the concentration of free receptor *R* is *R*_*max*_ *-R*, accordingly, the Eq (8) can be rearranged to:

$$\frac{dR}{dt}=k_{a}C_{L}(R_{max}-R)-k_{d}R$$ (9)

If the initial value *R*_*0*_ is 0 at the initial time *t*_*0*_ (*t*_*0*_ = 0), the value *R* can be solved from the Eq (9) using the Integral Transformation Method, which is written as the following expression illustrated at the arbitrary time *t*,

$$R=\frac{k_{a}R_{max}C_{L}}{k_{a}C_{L}+k_{d}}(1-e^{-k_{ob}t})$$ (10)

where, *k*_*ob*_ = *k*_*a*_ *C*_*L*_ *+k*_*d*_

Then, we use the value of *LR*_*eq*_ instead of $\frac{k_{a}R_{max}C_{L}}{k_{a}C_{L}+k_{d}}$, the Eq (10) can be expressed as,

$$R=LR_{\text{eq}}(1-e^{-k_{ob}t})$$ (11)

When the ligands combine with the receptors completely in the area of association, the dissociation process of compounds occurs. Therefore, in the dissociation process with the concentration of ligand *L* of 0, the Eq (9) can be rewritten in the following form.

$$\frac{dR}{dt}=-k_{d}R$$ (12)

For solving Eq (12) by Integral Transformation Methods, we get the values of RU from the experiment performed by the SPR biosensor, that is

$$RU=LR_{eq}e^{-k_{d}(t_{2}-t_{1})}$$ (13)

where, *t*_*1*_ is the initial time of dissociation, *t*_*2*_ is arbitrary time between the initial time and the end time, and RU is the response value of the SPR biosensor at time *t*_*2*_. The affinity constants can be determined from the data obtained from the SPR biosensor at the steady response state during the association phase.

Now, assume *y = RU*, *a = LR*_*eq*_ and *b = k*_*ob*_, the Eq (11) can be simplified as:

$$y=a(1-e^{-bx})$$ (14)

The kinetic model of dissociation process (13) can be simplified to

$$y=ae^{-\text{m}t}$$ (15)

where, *m* = *k*_*d*_. The value of *m* which is the dissociation rate constant calculated from the Marquardt algorithm was evaluated firstly. Then the association rate constant can be obtained in accordance with the expression *b = k*_*ob*_ = *k*_*a*_ *C*_*L*_+*k*_*d*_. Accordingly, the values of affinity constant *K*_*A*_ and *K*_*D*_ are calculated respectively.

## Establishment of Curve-Fitting Algorithms

### Gauss-Newton Algorithm

For the kinetic model of association *y* = *a*(1-*e* ^*-bx*^), the corresponding *y*_*i*_ were obtained from the experiment of the SPR biosensor. Once parameters *a*, *b* are obtained, the kinetic model of association for a particular biomolecular interaction can be formed successfully. In order to solve the equations, the initial value of *a*, *b* should be given, named *a*_*0*_, *b*_*0*_, respectively. The actual values of *a*, *b* were obtained from the following expressions:*a* = *a*_0_+Δ_1_ and *b* = *b*_0_+Δ_2_, where,Δ_1_,Δ_2_ represent the increments of *a*_0_, *b*_0_, respectively. Then the values of Δ_1_,Δ_2_ will be obtained from the following procedures. The function of *y* = *a*(1-*e* ^*-bx*^) is expanded using the Taylor series at the point (*a*_0_, *b*_0_). The results will be expressed in Eq (16) by ignoring the quadratic term.

$$y=y(a_{0},b_{0})+\frac{\partial y}{\partial a}\Delta_{1}+\frac{\partial y}{\partial b}\Delta_{2}=a_{0}(1-e^{-b_{0}x})+(1-e^{-b_{0}x})\Delta_{1}+(xa_{0}\text{exp}{\left(-b_{0}x\right)}^{-b_{0}x})\Delta_{2}$$ (16)

The residual value *Q* between experimental and theoretical value is obtained by utilizing least square method. The expression is shown as following,

Q=∑i=1N(yi−y)^2⇒Q=∑i=1N{yi−[a0(1−e−b0xi)+(1−e−b0xi)Δ1+(xia0e−b0xi)Δ2]}2 (17)

where, *y*(*a*_*0*_, *b*_*0*_) is determined by the known *a*_0_, *b*_0_. Both $\frac{\partial y}{\partial a}$ and $\frac{\partial y}{\partial b}$ are the function of independent variable *x*. Moreover, *x* is the experimental result. Hence, Eq (14) can be simplified to the linear relationship on Δ_1_,Δ_2_ as follows,

$$\frac{\partial Q}{\partial a}=\frac{\partial Q}{\partial \Delta_{1}}=-2\sum_{i=1}^{N}\left\{y-(a(1-e^{-b_{0}x_{i}})+(1-e^{-b_{0}x_{i}})\Delta_{1}+(-a_{0}x_{i}e^{-b_{0}x_{i}})\Delta_{2})\right\}\cdot (1-e^{-b_{0}x_{i}})=0$$ (18)

Rearrange above Eq (18) to the following Eq (19).

$$\begin{array}{l} \frac{\partial Q}{\partial \Delta_{1}}=\sum_{i=1}^{N}\left\{[y_{i}(1-e^{-b_{0}x_{i}})-{\left(a_{0}(1-e^{-b_{0}x_{i}})\right)}^{2}]-[{\left(1-e^{-b_{0}x_{i}}\right)}^{2}\cdot \Delta_{1}]+[(x_{i}a_{0}e^{-b_{0}x_{i}})\cdot \Delta_{2}-{\left(x_{i}a_{0}e^{-b_{0}x_{i}}\right)}^{2}\cdot \Delta_{2}]\right\}\\ \Rightarrow \sum_{i=1}^{N}\{[{\left(1-e^{-b_{0}x_{i}}\right)}^{2}\cdot \Delta_{1}]+[{\left(x_{i}a_{0}e^{-b_{0}x_{i}}\right)}^{2}-(x_{i}a_{0}e^{-b_{0}x_{i}})]\cdot \Delta_{2}\}=\sum_{i=1}^{N}\left\{[(1-e^{-b_{0}x_{i}})\cdot (y_{i}-a_{0}(1-e^{-b_{0}x_{i}}))]\right\} \end{array}$$ (19)

Correspondingly,

$$\frac{\partial Q}{\partial b}=\frac{\partial Q}{\partial \Delta_{2}}=-2\sum_{i=1}^{N}\left\{[y_{i}-a_{0}(1-e^{-b_{0}x_{i}})]+[(1-e^{-b_{0}x_{i}})\cdot \Delta_{1}]+[(-x_{i}a_{0}e^{-b_{0}x_{i}})\cdot \Delta_{2}]\right\}\cdot [(-x_{i}a_{0}e^{-b_{0}x_{i}})]=0$$ (20)

$$\frac{\partial Q}{\partial \Delta_{2}}=0\Rightarrow \sum_{i=1}^{N}\{[(x_{i}a_{0}e^{-b_{0}x_{i}})\cdot (1-e^{-b_{0}x_{i}})\Delta_{1}]+[{\left(x_{i}a_{0}e^{-b_{0}x_{i}}\right)}^{2}\cdot \Delta_{2}]\}=\sum_{i=1}^{N}\left[(x_{i}a_{0}e^{-b_{0}x_{i}})]\cdot [y_{i}-a_{0}(1-e^{-b_{0}x_{i}})\right]$$ (21)

where, the following substitution will be done.

$$\frac{\partial y}{\partial a}=(1-e^{-b_{0}x_{i}})=A,(i=1,2,3\ldots \ldots N)$$ (22)

$$\frac{\partial y}{\partial b}=(x_{i}a_{0}e^{-b_{0}x_{i}})=B,(i=1,2,3\ldots \ldots N)$$ (23)

$$\sum_{i=1}^{N}\left[(1-e^{-b_{0}x_{i}})\cdot (y_{i}-a_{0}(1-e^{-b_{0}x_{i}}))\right]=C$$ (24)

$$\sum_{i=1}^{N}\left[(x_{i}a_{0}e^{-b_{0}x_{i}})]\cdot [y_{i}-a_{0}(1-e^{-b_{0}x_{i}})\right]=D$$ (25)

So, both expressions (17) and expression (19) can be arranged to:

$$\left(\begin{array}{cc} \sum_{i=1}^{N}A^{2} & \sum_{i=1}^{N}AB\\ \sum_{i=1}^{N}AB & \sum_{i=1}^{N}B^{2} \end{array}\right)\cdot \left(\begin{array}{c} \Delta_{1}\\ \Delta_{2} \end{array}\right)=\left(\begin{array}{c} C\\ D \end{array}\right)$$ (26)

where

$$\left(\begin{array}{cc} \sum_{i=1}^{N}A^{2} & \sum_{i=1}^{N}AB\\ \sum_{i=1}^{N}AB & \sum_{i=1}^{N}B^{2} \end{array}\right)=\left(\begin{array}{ccccc} \frac{\partial y_{1}}{\partial a} & \frac{\partial y_{\text{2}}}{\partial a} & \ldots \ldots & \frac{\partial y_{N\text{-1}}}{\partial a} & \frac{\partial y_{N}}{\partial a}\\ \frac{\partial y_{1}}{\partial b} & \frac{\partial y_{\text{2}}}{\partial b} & \ldots \ldots & \frac{\partial y_{N\text{-1}}}{\partial b} & \frac{\partial y_{N}}{\partial b} \end{array}\right)\cdot \left(\begin{array}{cc} \frac{\partial y_{1}}{\partial a} & \frac{\partial y_{1}}{\partial b}\\ \frac{\partial y_{2}}{\partial a} & \frac{\partial y_{2}}{\partial b}\\ \ldots \ldots & \ldots \ldots \\ \frac{\partial y_{N\text{-}1}}{\partial a} & \frac{\partial y_{N-1}}{\partial b}\\ \frac{\partial y_{N}}{\partial a} & \frac{\partial y_{N}}{\partial b} \end{array}\right)$$ (27)

The expression (26) is the equation involving both unknown parameters Δ_1_,Δ_2_. Once both Δ_1_,Δ_2_ were obtained, both *a*_1_, *b*_1_ can be obtained according to the following expressions *a*_1_ = *a*_0_+Δ_1_, *b*_1_ = *b*_0_+Δ_2_. Here, *a*_0_,b_0_ are replaced by *a*_1_,b_1_. The iterative process may be continued to do until the criterion for convergence is satisfied (e.g. max |Δ_1_|< = *ε*_1_ and max |Δ_2_|< = *ε*_2_). However, this method is more dependent on the initial values. Obviously, different initial values can cause the iterative divergence. For this reason, the Marquardt algorithm was introduced.

### Marquardt algorithm

Marquardt algorithm is somewhat similar to the Gauss-Newton algorithm. Both the initial values *a*_0_, *b*_0_ are also previous given and the nonlinear model was implemented using the Taylor series expansion at the point (*a*_0_, *b*_0_). Both Δ_1_,Δ_2_ were figured out by conducting the tangential line. The iterative process may be continued to do until the situation for convergence is satisfied. In the Marquardt algorithm, residual value of *Q* is calculated by the following expression.

Q=∑i=1N(yi−y)^2⇒Q=∑i=1N{yi−(a0(1−e−b0xi)+(1−e−b0xi)Δ1+(−xiae−b0xi)Δ2)}2+dΔ12+dΔ22 (28)

Where,

$$\frac{\partial Q}{\partial \Delta_{1}}=0\Rightarrow (\sum_{i=1}^{N}A^{2}+d)\Delta_{1}+(\sum_{i=1}^{N}AB)\Delta_{2}=C$$ (29)

$$\frac{\partial Q}{\partial \Delta_{1}}=0\Rightarrow (\sum_{i=1}^{N}AB)\Delta_{1}+(\sum_{i=1}^{N}B^{2}+d)\Delta_{2}=D$$ (30)

Converted the above expression (30) into the matrix form,

$$\left(\begin{array}{cc} \sum_{i=1}^{N}A^{2}+d & \sum_{i=1}^{N}AB\\ \sum_{i=1}^{N}AB & \sum_{i=1}^{N}B^{2}+d \end{array}\right)\cdot \left(\begin{array}{c} \Delta_{1}\\ \Delta_{2} \end{array}\right)=\left(\begin{array}{c} C\\ D \end{array}\right)$$ (31)

where,

$$\left(\begin{array}{cc} \sum_{i=1}^{N}A^{2} & \sum_{i=1}^{N}AB\\ \sum_{i=1}^{N}AB & \sum_{i=1}^{N}B^{2} \end{array}\right)=\left(\begin{array}{ccccc} \frac{\partial y_{1}}{\partial a} & \frac{\partial y_{\text{2}}}{\partial a} & \ldots \ldots & \frac{\partial y_{N\text{-1}}}{\partial a} & \frac{\partial y_{N}}{\partial a}\\ \frac{\partial y_{1}}{\partial b} & \frac{\partial y_{\text{2}}}{\partial b} & \ldots \ldots & \frac{\partial y_{N\text{-1}}}{\partial b} & \frac{\partial y_{N}}{\partial b} \end{array}\right)\cdot \left(\begin{array}{cc} \frac{\partial y_{1}}{\partial a} & \frac{\partial y_{1}}{\partial b}\\ \frac{\partial y_{2}}{\partial a} & \frac{\partial y_{2}}{\partial b}\\ \ldots \ldots & \ldots \ldots \\ \frac{\partial y_{N\text{-}1}}{\partial a} & \frac{\partial y_{N-1}}{\partial b}\\ \frac{\partial y_{N}}{\partial a} & \frac{\partial y_{N}}{\partial b} \end{array}\right)$$ (32)

The procedure of Marquardt algorithm to solve parameters a, b is shown as following. (a) Initial values *a*_0_, *b*_0_ are given at first, and the corresponding initial value of squared residual *Q*_0_ was figured out. (b) Use iterative method to determine the parameter *d*. The initial value of *d* is set to be 0.01. Then, it was substituted into the expression (31) to calculate the value of Δ_1_,Δ_2_. The parameters *a*_1_, *b*_1_ and *Q*_1_ were obtained in the same approach mentioned above. Compare the value *Q*_1_ with *Q*_0_, if *Q*_1_>*Q*_0_, adjust the value *d* to repeat the above process until *Q*_1_<*Q*_0_. (c) Repeat to perform the above iterative process with these parameters *d*, *Q*_1_, *a*_1_, *b*_1_, until |Δ_1_|< = *ε* and |Δ_2_|< = *ε* (*ε* = 10^−6^). Finally, the coefficients *a*, *b* of the kinetic model function *y* = *a*(1-*e* ^*-bx*^) were achieved.

## Experimental Validation for the Biomolecular Interactions between HBsAb and HBsAg

Retention of binding activity is the most important consideration when immobilizing a biomolecule on the Au film of SPR biosensor and can be measured by comparing the relative binding responses recorded as RU. (a) Immobilizing the HBsAb on the surface plasmon resonance sensor surface, then the PBS (pH 7.4) was used to clean the sensor surface in order to obtain a highly stable response baseline. (b) Flowing of sample solution of HBsAg diluted to 100-fold through the microfludic cell (estimated concentration 16ng·mL^-1^), and monitoring the binding of HBsAg to immobilized HBsAb on the Au film deposited on the SPR biosensor, it causes a modification of the refractive index at the surface and results a change of the resonant angle. After a certain reaction time, an equilibrium plateau was reached, where there are no changes of signals produced with time. We injected the PBS to the microfludic cell to block the association of HBsAg-HBsAb compounds. At this time, the association between HBsAg and HBsAb was not existed, so the concentration of HBsAg was 0. (c) The HCL (pH 3.0) solution was used to remove all the HBsAg molecules to regenerate the SPR biosensor. Then, the buffer solution PBS was injected to restore the baseline again and a new cycle was beginning.

## Results and Analysis

**Fig 1:**
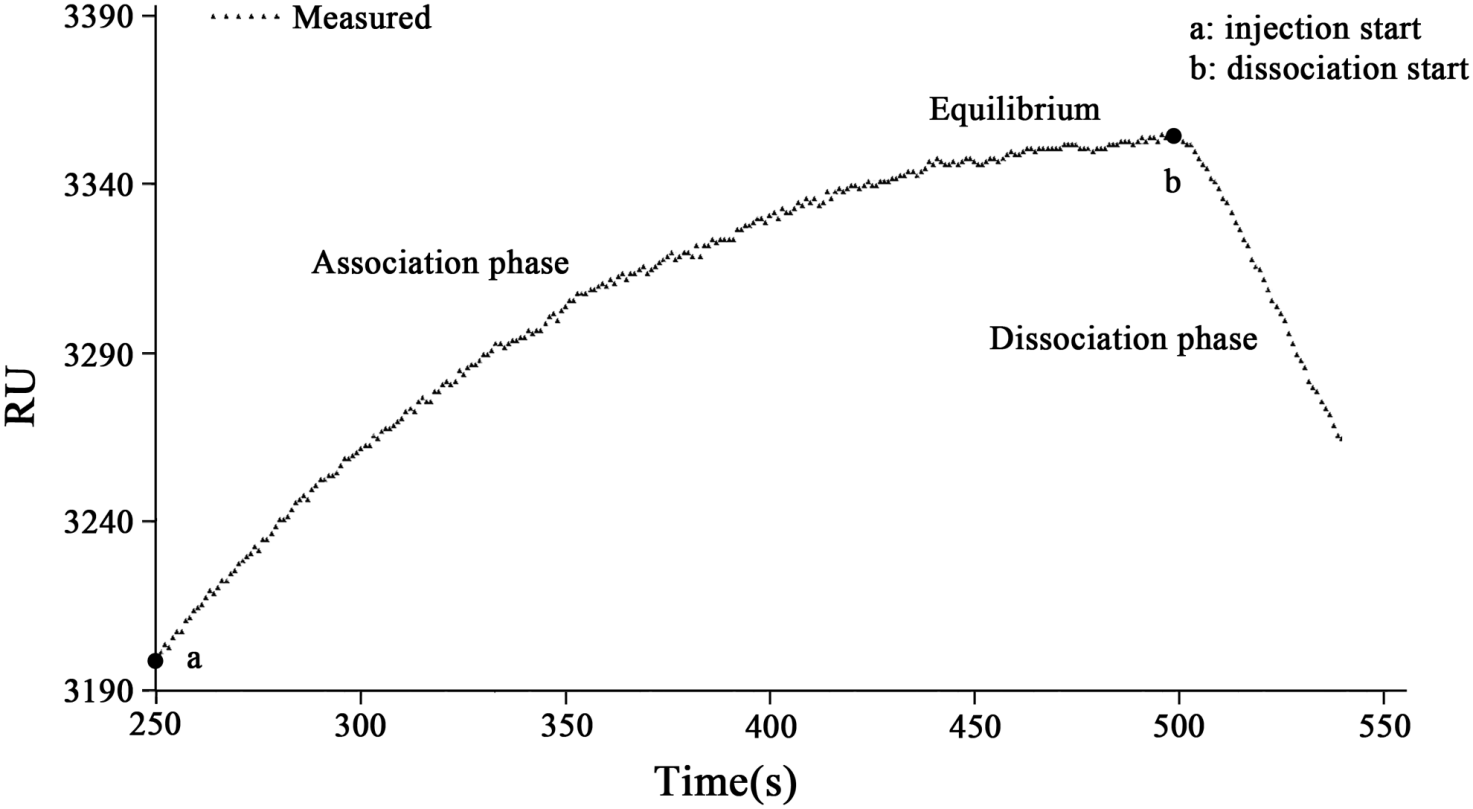
Sensorgram showing the association and dissociation processes of biomolecular interaction between HBsAg and HbsAb. The data marked with a triangle is obtained in the average of more than three sets of measurement results in RU. This sensorgram is showing that the HBsAg was binding on the specific HBsAb (association phase) starting from the injection point a and reaches an equilibrium after approximately 251s. From the dissociation starting point b, the dissociation phase was formed sequentially. The microfludic cell of this SPR bioanalyzer was kept at a constant temperature of 37°C.

The parameters and the initial values can be set manually according to the experimental data in the SPR biomolecular interaction analysis software based on the Marquardt algorithm, which was designed by our research group. Different results from various initial values can be compared each other visually so that we can select the most ideal initial values to reduce errors. From the homemade SPR analysis software, the experimental data can be imported conveniently. The x-axis of the graph represents time (s), while the y-axis of the graph represents the signal responses indicated with RU, which was computed based on the following formula RU = (1.334-RIx) ×30000, where 1.334 was the refractive index of deionized water. RIx is the refractive index of an unknown sample, which can be measured by using the SPR biosensor in real-time and 30,000 is a pre-determined factor for increasing the sensitivity of the calculated responses [22]. The initial time was set to be 250s, and the time of association phase and dissociation phase were 251s and 38s. Fig 1 is the response curve for the sequential injection of HBsAg solution, indicating the association phase and dissociation phase of HBsAg and HBsAb. The dotted lines mark the injection of the HBsAg solution with RU (response unit) values.

**Fig 2:**
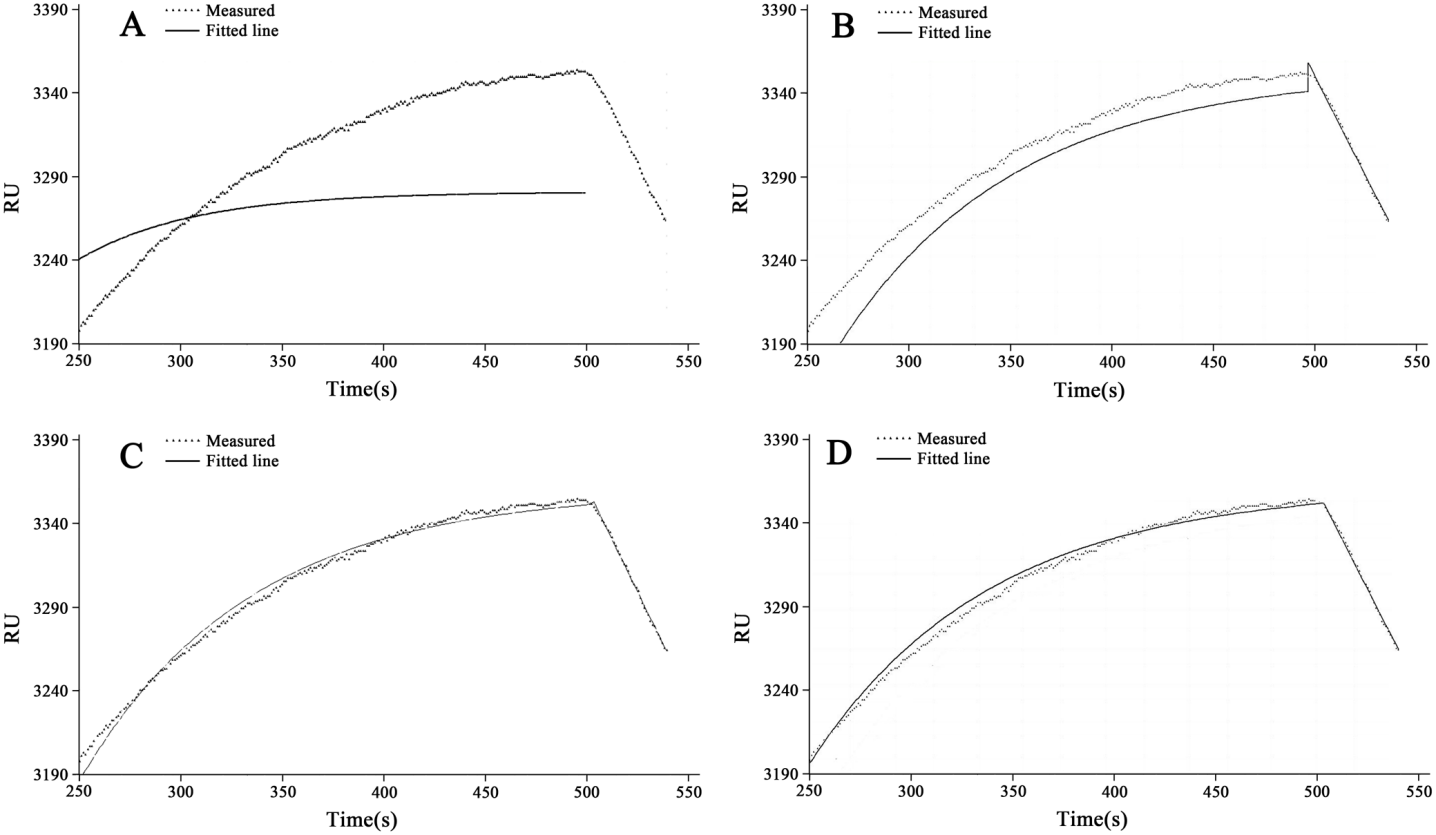
The fitting results obtained from both Newton Iteration algorithm and Marquardt algorithm. The data marked with a triangle is obtained in the average of more than three sets of measurement results in RU, and the fitted curve was marked with a solid line. A. The curve-fitting using the Newton Iteration algorithm with the initial value of 0.0095, B. The curve-fitting using the Newton Iteration algorithm with the initial value of 0.011, C. The curve-fitting using the Marquardt algorithm with the initial value of 0.0095, D. The curve-fitting using the Marquardt algorithm with the initial value of 0.011.

The curve-fitting obtained from using both Newton Iteration algorithm and Marquardt algorithm was shown in Fig 2.

In sub-figures of Fig 2, the dotted lines represent the original data collected from the data acquisition system of this homemade bioanalyzer designed by using the homemade SPR bioanalyzer [22], while the solid lines represent the nonlinear curve-fitting to match the affinity kinetic model. From Fig 2, the following conclusions can be obtained: (1) The fitting results obtained from the Newton Iteration algorithm is divergent to some non-proper initial values (Fig 2A and 2B). However, Marquardt algorithm can avoid this problem effectively; (2) In the same initial conditions, the results obtained from the Marquardt algorithm had much better than the results obtained from the Newton iteration algorithm (Fig 2C and 2D).

In this experiment, the introductions of damping coefficient in Marquardt algorithm can real-time amend the increments of parameters so that the possibility of divergence is greatly reduced. The experimental data was obtained from HBsAg biomolecules with concentration of 16ng·mL^-1^. The curve-fitting is shown in sub-Figured in Fig 2 and the *a* = 3358.23246, b = 0.01188 in the association phase, a = 3358.73684, m = -0.00073 in the dissociation phase were obtained, respectively. Hence, the *R*_*max*_, *k*_*a*_, *k*_*d*_ *K*_A_, and *K*_*D*_ are estimated according to the data narrated above (See Table 1).

**Table 1:** Kinetic constants of molecular interaction between HBsAg and HBsAb.

Fitting curves	Kinetic models	Kinetic constants
Association process	$RU=LR_{\text{eq}}(1-e^{-k_{ob}t})$	*LR_eq_* = 3358.232, *k_ob_* = 0.01188 s^-1^, *R*_max_ = 3559.486, *C_L_* = 16 ng· mL^-1^
Dissociation process	$RU=LR_{\text{eq}}e^{-k_{d}(t_{2}-t_{1})}$	*k_d_* = 0.00073 s^-1^, *K_D_* = 1.0475×10^−9^ g·mL^-1^, *k_a_* = 6.969×10^5^ mL·g^-1^·s^-1^, *K_A_* = 9.5466×10^8^ mL·g^-1^

## Conclusions

Surface plasmon resonance biosensors are important tools in characterizing biomolecular interactions as well as understanding the biomolecular recognition membrane established on the surface of the Au film of the SPR biosensor. A number of applications using SPR biosensors have been found in food safety, environmental pollutants and life science, which were related to the experimental design and the calculation of kinetic constants involving the biomolecular interaction process between antigen and antibody or receptors and ligand. To understand clearly the interaction process, we confirms that the data does indeed obey the pseudo-first-order binding interaction model and validates the extracted kinetic and affinity constants. This article has established an approach based on Marquardt algorithm for the analysis of the biomolecular interaction using an optical surface plasmon resonance biosensor. The Marquardt algorithm was addressed experimentally to provide better understanding the results compared to the Newton iteration algorithm with reducing the possibility of divergence when the curve fitting was established. A global fitting of the dissociation rate constant (*k*_*d*_) was performed firstly. The next global fitting with *ka* and *Rmax* (fixed *k*_*d*_ as a constant) was sequential obtained. The association and dissociation rate constants *ka* and *k*_*d*_ were 6.969×10^5^ mL·g^-1^·s^-1^ and 0.00073 s^-1^, giving an affinity constant (*K*_*D*_) of 1.0475×10^−9^ g·mL^-1^ from the HBsAg with concentration of 16ng·mL^-1^, respectively. With the careful curve-fitting, surface plasmon resonance biosensors may be applied to provide accurate kinetic constants.
